# Proceedings of the Georgia State Dental Society

**Published:** 1875-03

**Authors:** 


### ARTICLE III.

### *Georgia State Dental Society.*

The sixth annual meeting of this Society convened in Atlanta, the 11th, and adjourned the 14th of May. Dr. A. C. Ford, of Atlanta, President, in the chair.

Sixteen new members were added to the rolls. The following papers were read:

Dental Histology and Physiology, by Dr. George Peterson, of Waynesboro. Dental Surgery, by Dr. E. Parsons, of Savannah. Mechanical Dentistry, by Dr. J. L. Fogg, of

Barnesville. Dr. James A. Hart, of Hawkinsville, read a description of a case of surgery in his practice, and exhibited some rare specimens of abnormal growth of teeth.

The Executive Committee chosen by the Society, and the law passed by the State Legislature, to regulate the practice of dentistry in the State, reported a form and method of granting license which the Society approved.

Resolutions upon the deaths of Drs. Fogle, of Columbus, (one of the oldest members in the State,) and Campbell, of Atlanta, were read, and spread upon the minutes.

Officers elected for the ensuing year:—

*President.*—Dr. George Peterson, of Waynesboro.

1*st. Vice-President.*—Dr. G. W. McIlhaney, of West Point.

2*nd Vice-President.*—Dr. J. P. Holmes, of Macon.

*Corresponding Secretary.*—Dr. M. S. Jobson, of Perry.

*Recording Secretary.*—Dr. L. D. Carpenter, of Atlanta.

*Executive Committee.*—Dr. E. Parsons, of Savannah; E. M. Allen of Marietta; J. P. H. Brown, of Augusta; W J. Tigner, of Columbus; and H. A. Lowrance, of Athens.

Committees to report at next meeting:—

*Histology and Physiology.*—Drs. Chas. C. Allen, of Marietta, and D. S. Wright, of Macon.

*Pathology and Surgery.*—Drs. J. P. H. Brown, of Augusta, and M. H. Thomas, of Monroe.

*Chemistry and Therapeutics.*—Dr. L. S. Ledbetter, of Cedartown, and D. Parsons, of Savannah.

*Operative Dentistry.*—Drs. A. C. Ford, of Atlanta; and M. S. Jobson, of Perry.

*Mechanical Dentistry.*—Drs. S. J. Holland, of Atlanta, and R. J. Hampton, of Rome.

*Dental Education and Literature.*—Drs. F. Y. Clarke, of Savannah, and J. H. Coyle, of Thomasville.

*Clinical Operators.*—Drs. L. D. Carpenter, of Atlanta; R. W. Thornton, of Calhoun; J. W. Murrell, of Athens, and J. P. Holmes, of Macon.

Dr. G. F. S. Wright, of South Carolina, by request exhibited the Electrical Burring Engine and Mallet.

Clinics were held by Drs. Wright, of South Carolina; Pool, of Columbus, and Ford, of Atlanta.

The next annual meeting will be held in Atlanta, (as provided by the Constitution,) on the second Monday in May next, (10th,) at 10 o'clock A. M. All dentists in the State particularly invited to be present, and all from other States who are in good standing, cordially invited to attend.

